# The Development and Use of Office of New Drugs Custom Medical Queries for Safety Analyses of Clinical Trial Data

**DOI:** 10.1007/s40264-025-01582-1

**Published:** 2025-09-15

**Authors:** Scott Proestel, Vaishali Popat, Ellis F. Unger, Linda J. B. Jeng

**Affiliations:** 1 Division of Biomedical Informatics, Research, and Biomarker Development, Office of Drug Evaluation Sciences, Office of New Drugs, Center for Drug Evaluation and Research, FDA, 10903 New Hampshire Ave., Building 22, Room 5215, Silver Spring, MD 20093 USA; 2 Division of General Medicine, Office of Clinical Evaluation, Office of Therapeutic Products, Center for Biologics Evaluation and Research, FDA, Silver Spring, MD USA; 3 Hyman, Phelps & McNamara, P.C., Washington, DC USA

## Abstract

The evaluation of safety data by the US Food and Drug Administration (FDA) is a critical step in the review of marketing applications for drugs and biologics. It can be difficult to identify a safety signal, and important signals can be missed if not evaluated comprehensively. Adverse events reported by study participants constitute a major source of safety data, and while previously established standard term groupings have been useful for analysis (e.g., Standardised MedDRA^®^ Queries), the Office of New Drugs (OND) at the FDA determined a need for more clinically meaningful groupings specifically designed for use in premarket drug safety evaluation. To improve safety signal detection and analyses of adverse reactions, OND developed standard groupings of adverse event terms known as OND Custom Medical Queries (OCMQs). OCMQs are intended to capture clinically meaningful groupings (i.e., safety signals) represented in premarketing data. OND has seen great utility in OCMQs during premarket drug safety evaluations, as they have improved OND’s ability to detect safety signals and to distinguish and quantify adverse reactions in clinical trial data.

## Key Points

**Table:** 

There is a need for standard adverse event term groupings designed specifically for use in premarket drug safety evaluation by the Office of New Drugs (OND).
The OND has developed a specialized term grouping system for adverse event analysis known as OND Custom Medical Queries (OCMQs).

## Introduction

The evaluation of safety data by the US Food and Drug Administration (FDA) is one of the most critical functions in the review of marketing applications for drugs and biologics.^1^ It can be difficult to identify a safety signal, and important signals can be missed if not evaluated comprehensively. Adverse events (AEs) reported by study participants to investigators constitute one of the major sources of safety data in marketing applications. Typically, investigators record symptoms or complaints from study participants in their own words as “verbatim terms.” Verbatim terms provide the crucial link between study participants and AE analyses, but they may include shorthand, misspellings, and additional detail not needed for coding (e.g., “L hip pain,” “painful right hip,” and “hip pain w walking”). Therefore, verbatim terms are matched to a standard term using a standardized terminology known as the Medical Dictionary for Regulatory Activities (MedDRA^®^) [1]. The standard terms provided by MedDRA^®^ are known as Preferred Terms (PTs). For example, the above verbatim terms would all be coded to the PT *Pain in hip*.

In some cases, MedDRA^®^ adds specificity to PTs that may be counterproductive with respect to AE analyses. For example, there are numerous PTs for insomnia. For a study participant with difficulty falling asleep, an investigator might reasonably record the verbatim term “insomnia,” whereas another investigator might record “trouble falling asleep.” These verbatim terms would likely be coded to the PTs *Insomnia* and *Initial insomnia*, respectively. Upon tabulation of individual PTs, these terms would be separated and result in an underestimation of the incidence of insomnia. In another example, PTs of *Lethargy*, *Listless*, and *Fatigue* are each in a separate MedDRA^®^ System Organ Class (SOC) (*Nervous system disorders*, *Psychiatric disorders*, *General disorders and administration site conditions*, respectively), but all three terms might be grouped in a custom query to improve signal detection. The separation of related PTs has been a longstanding concern, as previously noted in the FDA’s March 2005 “Guidance for Industry: Premarketing Risk Assessment,” which states that “Events that are reported under different terms in the database, but that represent the same phenomenon (e.g., sedation, somnolence, drowsiness) should ordinarily be grouped together as a single adverse reaction (AR) to avoid diluting or obscuring the true effect” [2]. While some AE groupings existed at that time (e.g., the MedDRA^®^ hierarchy and Standardised MedDRA^®^ Queries [SMQs]), the Office of New Drugs (OND) determined that there was a need for more clinically meaningful groupings specifically designed for use in premarket drug safety evaluation.

## Office of New Drugs Custom Medical Queries (OCMQs)

To help identify potential safety signals during review of clinical trial AE data, OND developed 104 custom groupings of AE terms known as OND Custom Medical Queries (OCMQs) that are focused on commonly labeled ARs, as well as medically important but less common reactions that have historically been drug related (Table 1). This custom grouping was presented at the Duke-Margolis Center for Health Policy Public Workshop: Advancing Premarket Safety Analytics [3]^2^.

**Table 1:**
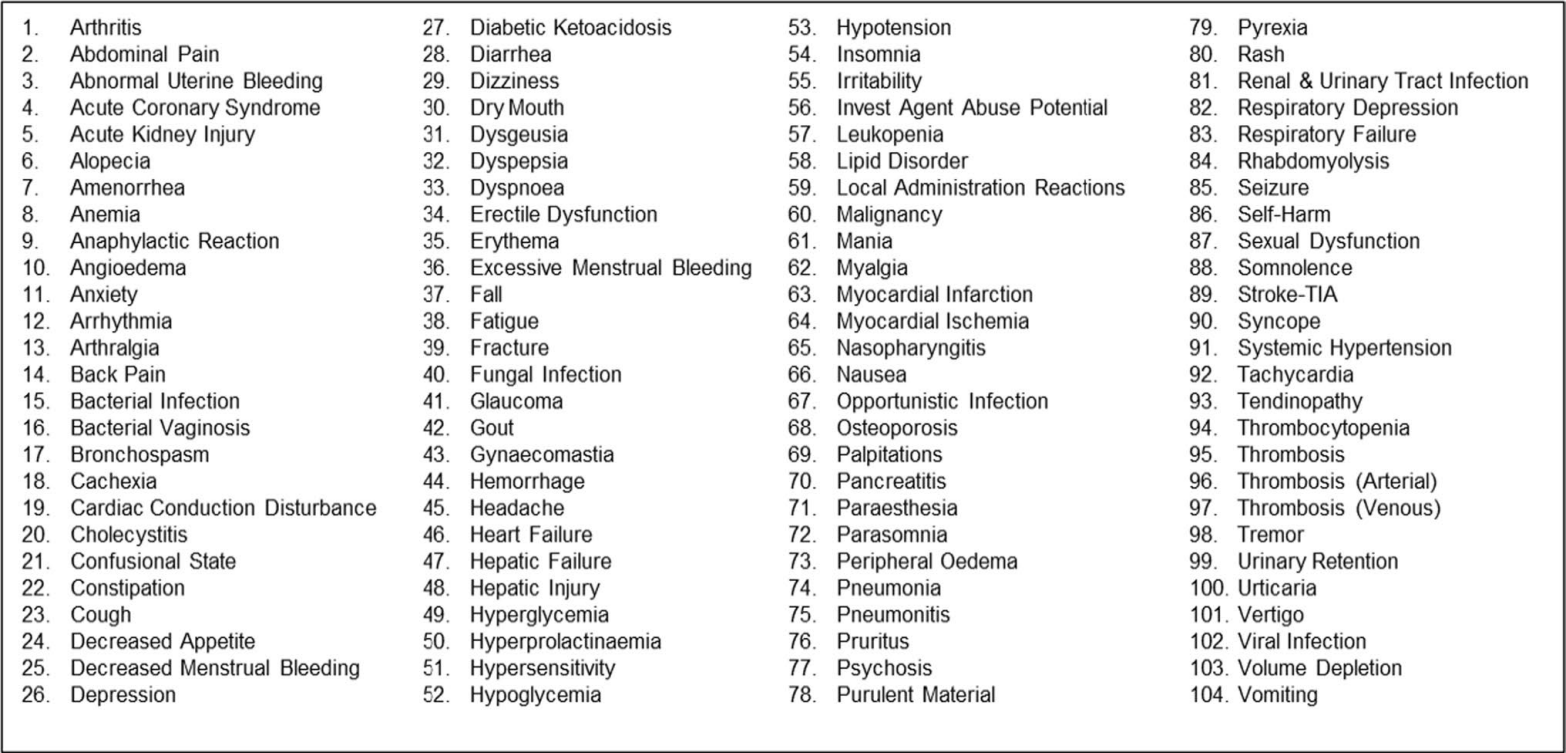
OND custom medical queries version 2.1

Current and future versions of the OCMQs can be accessed at the FDA OCMQ webpage [4]

*FDA* Food and Drug Administration, *OCMQ* OND Custom Medical Query, *OND* Office of New Drugs

*Methods:* The most frequently encountered ARs were identified using natural language processing of more than 38,000 labels from 1960 to 2017,^3^ as well as additional terms that identify medically important events historically known to be drug related. In phase 1 of development, 60 of the most frequently occurring terms were identified. In phase 2, all review divisions were contacted to generate a list of queries that reviewers found useful for their specific therapeutic areas. Phase 3 included development of Algorithmic OCMQs. Several working groups were created, and each query was developed by a group of six to eight reviewers. Three reviewers independently categorized related MedDRA^®^ terms into a Narrow and a Broad OCMQ. A database of AE terms derived from over 5000 clinical trials formed the basis of potential terms to categorize. In addition, the following resources were used to help identify terms to include in the OCMQs:

1. MedDRA^®^ hierarchy
2. MedDRA^®^ browser
3. Existing queries
4. Frequently co-occurring PTs in the FDA Adverse Event Reporting System (FAERS) database

Draft OCMQs were shared broadly within the Agency to solicit input and feedback. If there was a discrepancy in categorization of PTs among two independent reviewers, the working group decision was considered final.

OCMQs are intended to capture clinically meaningful groupings (e.g., safety signals) represented in premarketing data. For example, to assess the true incidence of insomnia in a clinical trial, the OCMQ *Insomnia* was developed and includes PTs such as *Initial insomnia*, *Middle insomnia*, and *Early morning awakening*. These PTs, along with others, need to be combined to accurately assess the incidence of insomnia in a clinical trial. It is important to note that OCMQs do not include all MedDRA^®^ PTs, so review of individual reported PTs during clinical trial data safety analysis is still necessary.

The development of OCMQs was the product of the medical judgment of more than 80 FDA clinical reviewers from most of the review divisions within FDA’s Center for Drug Evaluation and Research (CDER), with additional representation and input from FDA’s Center for Biologics Evaluation and Research (CBER). OCMQ Ground Rules were adopted to maintain consistency in the application of medical judgment to the AE groupings (Table 2).

**Table 2:** OCMQ ground rules

Narrow	Broad
1. AEs that are near synonyms of the OCMQ (e.g., PT *Abdominal discomfort* in OCMQ *Abdominal Pain*) 2. AEs that are subgroups of the OCMQ (e.g., PT *Anaemia neonatal* in OCMQ *Anemia*) 3. AEs that specify an etiology for the OCMQ (e.g., PT *Uraemic pruritus* in OCMQ *Pruritus*) 4. AEs that ensure the occurrence of the OCMQ (e.g., PT *Aortic rupture* in OCMQ *Hemorrhage*)	1. AEs that may indicate the presence of the OCMQ (e.g., PT *Osteopenia* in OCMQ *Osteoporosis*) 2. AEs that provide laboratory, radiologic, or other diagnostic test results reasonably suggestive of an OCMQ, including AEs with ambiguous results such as “abnormal” (e.g., PT *Blood glucose abnormal* in OCMQ *Hyperglycemia*) 3. AEs reasonably suggestive of the OCMQ, but not required by the OCMQ (e.g., PT *Bronchospasm* in OCMQ *Hypersensitivity*) 4. AEs that indicate a “carrier” status for OCMQs that specify an infectious disease (e.g., PT *Bacterial disease carrier* in OCMQ *Bacterial Infection*)

*AE* adverse event, *OCMQ* OND Custom Medical Query, *OND* Office of New Drugs, *PT* Preferred Term

The OCMQ Ground Rules establish two types of AE groupings: Narrow and Broad. The Narrow category terms are designed to provide a high degree of specificity. When an AE from an OCMQ Narrow grouping is reported, there is a high likelihood that the medical condition represented by the OCMQ term has occurred. The OCMQ Narrow groupings aim to ensure a high level of confidence in the association between the AE and the OCMQ Narrow grouping. For instance, the PT *Appendiceal abscess* is included in the OCMQ *Bacterial Infection*, because if a study participant experienced an appendiceal abscess, there is a very high likelihood they experienced a bacterial infection. The Broad category terms are designed to be more inclusive than the Narrow terms, providing greater sensitivity at the cost of specificity. When an AE from an OCMQ Broad grouping is reported, it suggests that the medical condition represented by the OCMQ term has occurred. The OCMQ Broad groupings aim to enhance the detection sensitivity of the OCMQ Narrow grouping. During development of the OCMQs, quantification of likelihoods was not plausible; assignment to Narrow or Broad categories necessarily involved medical judgment.

In addition to the OCMQ Ground Rules used to identify Narrow and Broad AE terms, the following rules were also used to exclude terms unlikely to be helpful in safety signal detection:

1. AEs that are neither a required component nor reasonably specific for the OCMQ are excluded (e.g., PT *Nausea* would not be included in OCMQ *Migraine*).
2. AEs that provide the names of laboratory, radiologic, or other diagnostic tests without a result are excluded (e.g., PT *Clostridium test*). AEs that provide test names without a result but that would only be performed in the presence of disease are included if they otherwise qualify (e.g., PT *Antipsychotic drug level* in OCMQ *Psychosis* [Broad]).
3. AEs that identify congenital, familial, or genetic disorders are excluded.^4^
4. AEs that identify pregnancy, puerperium, and perinatal conditions are excluded.^5^

An example of a partial listing of an OCMQ is displayed in Table 3, which provides many of the terms in the OCMQ *Depression*. Of note, all of the Narrow terms include the word “depression.” While word inclusion is not a requirement for the Narrow category, the terms in the table highlight the high degree of certainty required for inclusion. For example, the PT *Aortic rupture* does not contain information regarding bleeding or hemorrhage but is included in the Narrow OCMQ *Hemorrhage* term because it is clear that aortic rupture would cause hemorrhage.

**Table 3:** OCMQ example—*Depression* (partial listing)

Narrow terms	Broad terms
Agitated depression Childhood depression Depression Depression postoperative Depression suicidal Major depression Menopausal depression Persistent depressive disorder	Apathy Crying Decreased interest Dysphoria Feeling guilty Feeling of despair Feelings of worthlessness Helplessness Self-injurious ideation Suicidal behavior Suicidal ideation Suicide attempt

*OCMQ* OND Custom Medical Query, *OND* Office of New Drugs

As noted above, AEs included in the Broad OCMQ category are suggestive that the medical condition indicated by the OCMQ term occurred. As can be seen in Table 3, the terms included within the Broad category are highly suggestive of the OCMQ. The Broad category is not anticipated to lead directly to regulatory action but may serve as the basis for requesting additional information or pursuing additional analyses.

## Difference from Existing Groupings

The purpose of developing the OCMQs was to create clinically meaningful groupings of AEs for use by OND staff to promote a consistent safety signal detection strategy for premarket analysis. Existing groupings were not designed with this intent. For example, with respect to the MedDRA^®^ hierarchy, there are High Level Terms (HLTs), High Level Group Terms (HLGTs), and SOCs that can be used to group PTs. However, MedDRA^®^ terminology covers a much broader range of terms than AEs, such as therapeutic indications, investigations, product quality issues, medical procedures, and medical and social family history characteristics. Perhaps more importantly, MedDRA^®^ hierarchy group PTs use multiple grouping strategies (e.g., based on anatomy, pathology, physiology, etiology, manifestation site, purpose, or function).

Given the different strategies used, it is not surprising that different groupings have been produced. For example, an OCMQ for *Abdominal Pain* would require combining PTs from at least two HLTs within the MedDRA^®^ hierarchy (i.e., *Gastrointestinal and abdominal pains [excl oral and throat]* and *Gastrointestinal signs and symptoms not elsewhere classified [NEC]*). Table 4 provides a partial listing of these HLTs, with PTs considered suitable for inclusion in the OCMQ *Abdominal Pain* underlined. One can see multiple examples of PTs that are not consistent with abdominal pain, such as *Breath odour* and *Hiccups*. Therefore, use of the MedDRA^®^ hierarchy in this example would require inclusion of multiple terms unrelated to the OCMQ, which could obscure a safety signal. To be comprehensive and inclusive, OCMQs contain terms from the MedDRA^®^ hierarchy and other terms that have been submitted in the safety data of marketing applications, including misspelled terms identified in clinical trial datasets, based on manual review of combined datasets from more than 5000 clinical trials.

**Table 4:** MedDRA^®^ hierarchy example: PTs in the OCMQ *Abdominal Pain* (underlined)

HLT *Gastrointestinal and abdominal pains (excl oral and throat)* (partial listing)	HLT *Gastrointestinal signs and symptoms NEC* (partial listing)	
PTs Abdominal migraine Abdominal pain Abdominal pain lower Abdominal pain upper Abdominal rebound tenderness Abdominal rigidity Abdominal tenderness Gastrointestinal pain Infantile colic Oesophageal pain Visceral pain	PTs Abdominal discomfort Abdominal symptom Acute abdomen Anal incontinence Bradyphagia Breath odour Bruxism Cullen’s sign Dumping syndrome Dysphagia Dysphagia lusoria Early satiety Encoporesis Fixed bowel loop Foetor hepaticus Gastrocardiac syndrome Gastrointestinal somatic symptom disorder Gastrointestinal wall thickening Gastrointestinal wall thinning Hiccups	PTs Hyperphagia Hypophagia Incontinence Intestinal calcification Intestinal congestion Malignant dysphagia Mastication disorder Merycism Myochosis Oesophageal discomfort Oesophageal food impaction Pelvic discomfort Pelvic pain Peripancreatic fluid collection Peristalsis visible Pharyngeal dystonia Portal venous gas Post cholecystectomy syndrome Radiation dysphagia Radiation sickness syndrome White nipple sign Wischnewsky spots

*HLT* High Level Term, *MedDRA*^*®*^ Medical Dictionary for Regulatory Activities, *NEC* not elsewhere classified, *OCMQ* OND Custom Medical Query, *OND* Office of New Drugs, *PT* Preferred Term

SMQs are a widely used strategy for grouping PTs. A key distinction is that SMQs may exclude PTs that are presumed to be not drug related, while OCMQs attempt to capture all instances of a medical concept, regardless of the etiology or potential to be drug related. For example, the OCMQ *Pancreatitis* includes the PTs *Alcoholic pancreatitis*, *Autoimmune pancreatitis*, *Obstructive pancreatitis*, and infectious causes of pancreatitis such as *Pancreatitis viral*, whereas the SMQ version for *Acute pancreatitis* excludes these PTs.

There are several reasons why we believe it is appropriate to include all instances of a medical concept. First, the etiology specified by the AE may represent the mechanism by which a medication causes an AR. For instance, even if no current medications are known to cause a certain AR, novel therapeutics are continually under development with new mechanisms of action and potential off-target effects, and we do not believe that it is appropriate to make assumptions regarding drug effects. Another reason for including AEs that specify an etiology is that study participants experiencing those AEs might be more susceptible to a medication that causes the AR. For example, study participants with a history of alcoholic pancreatitis who receive such a diagnosis during a clinical trial might represent a group that is particularly susceptible to a medication that causes pancreatitis due to their pre-existing impairment. Therefore, an analysis that includes all pancreatitis-related AEs may increase the likelihood of detecting a safety signal in the pre-market setting. Last, clinical trial investigators may misattribute the cause of an AE. Using alcoholic pancreatitis again as an example, the occurrence of pancreatitis during a clinical trial in a participant with a history of alcoholic pancreatitis might lead an investigator to incorrectly attribute the event to alcoholic pancreatitis. It is important to consider all related AEs regardless of assessed causality to comprehensively evaluate data for safety signals.

## Algorithmic OCMQs

Algorithmic OCMQs use a set of rules that leverage additional information, such as combinations of AEs, laboratory data, concomitant medications, medical history, or timing information, to identify safety signals. Four Algorithmic OCMQs have been developed so far: *Muscle Injury*, *Hyperglycemia*, *Hypoglycemia*, and *Hypersensitivity*. These OCMQs contain Narrow and Broad terms similar to other OCMQs, but also use combinations of AEs, laboratory data, and timing information to make better use of the clinical trial data in identifying safety signals. Requiring the co-occurrence within the same study participant of otherwise non-specific AEs may enable the identification of ARs that were not identified during the trial. Likewise, combining laboratory and AE data is expected to provide a more comprehensive view of the AEs occurring during a trial.

## OCMQ Validation

The development of OCMQs was based primarily on clinical expert opinion. Therefore, we assessed whether OCMQ version 2.1 performed as expected when used to capture safety signals in pre-market clinical trial data. To investigate this issue, success was defined as a risk ratio (RR) > 1, where the numerator consisted of the overall OCMQ incidence for study participants who received an investigational drug labeled for the OCMQ of interest, and the denominator consisted of the overall OCMQ incidence for study participants in the control arm who did not receive the investigational drug labelled for the OCMQ of interest. For OCMQs performing as expected, the RR was expected to be > 1, as more study participants were expected to have experienced the OCMQ when receiving drugs for which the OCMQ was a known AR.

A total of 459 unique clinical trials were identified within the FDA’s Electronic Document Room using rules such as requiring the data to be in Clinical Data Interchange Standards Consortium (CDISC) format to improve interpretability, using only phase 3 trials, as they typically serve as the basis for marketing approval and evaluation of safety, and not using integrated datasets, to avoid counting study participants more than once. OCMQ incidence rates for each trial arm for all the trials were then calculated. A total of 93% of the Narrow OCMQs, 94% of the Broad OCMQs, and 75% of the Algorithmic OCMQs had an RR > 1 and were therefore considered to be performing as intended. The OCMQs with an RR ≤ 1 were further evaluated to understand the drivers of performance. As a result of this assessment, adjustments to some of the OCMQs were recommended and may be incorporated in a future OCMQ version.

This validation demonstrated that OCMQs are broadly successful at detecting signals in clinical trial data for drugs that are labeled for ARs represented by the OCMQs. The strength of this validation was the large database of clinical trial safety data used for the validation work. An important limitation was that the OCMQs were not tested in the specific manner for which they are intended to be used. Specifically, when a difference is detected in an OCMQ grouping between control and treatment arms, reviewers are expected to assess the individual AE terms within the OCMQ to determine whether there are a limited number of terms driving the disparity, a more subtle imbalance across many terms, or a combination of both. Each of these potential outcomes may lead to a different interpretation of the safety data, which requires clinical judgment. In addition, while the difference in AE incidence is an important consideration when assessing safety signals, other information is also important such as biologic plausibility, time to event, and AE severity. However, during this validation we used only the overall measure of AE incidence provided by the OCMQ groupings as it was not feasible to perform full safety reviews for all 459 trials analyzed.

In summary, this validation demonstrated that OCMQs are broadly successful at detecting signals in clinical trial data for drugs and biologics. Minor revisions to the OCMQs were recommended by clinical reviewers to enhance consistency with the OCMQ Ground Rules. Additional validation work will be considered, including assessment of the differences in signal detection when using only individual AEs compared to using OCMQ groupings and investigation of the use of OCMQs by review teams during the conduct of clinical reviews.

## Implications for Labeling

When a safety signal is identified using an OCMQ, it is important to note that the regulatory standard for including ARs in drug labels remains the same. Thus, if the results of an OCMQ analysis lead to a conclusion that there is an AR, medical and regulatory judgement should be used to determine whether it is appropriate to list some or all of the individual AEs of the OCMQ in the AR table or simply list the OCMQ name in the label. If the OCMQ name is included in the table and individual AEs of the OCMQ qualify as ARs, the individual AEs should be listed either in the table indented under the OCMQ or included as a table footnote.

## Conclusion

Although there is still much work to be done in support of OCMQ development, we have seen utility in using OCMQs in our premarket drug safety evaluations and anticipate that further improvement and implementation of the OCMQ strategy described in this report will improve OND’s ability to detect safety signals and to distinguish and quantify ARs in clinical trial data.

## Footnotes

1 For the remainder of this article, both drugs and biologics will be referred to as drugs.

2 Renamed from FDA Medical Queries (FMQs) in January 2025.

3 Combination products were excluded and only one label per active moiety was included.

4 While it is possible for such disorders to first become clinically apparent because of a drug effect during a clinical trial, such diagnoses in clinical trials are extremely rare and their manifestation as an AR to a drug rarer still. In addition, many of these diagnoses have heterogeneous manifestations and variable presentations that are difficult to categorize into OCMQs and are poorly characterized due to their infrequent occurrence. Any congenital, familial, or genetic disorders reported during a clinical trial can be identified using the MedDRA^®^ SOC for these terms.

5 Such conditions are quite rare in a typical clinical trial, and when they do occur, they should be carefully analyzed. For trials that specifically investigate pregnancy or breastfeeding, that allow these as part of their eligibility criteria, or that include neonatal or infant populations, the *Pregnancy, puerperium and perinatal conditions* MedDRA^®^ SOC can be used to identify these terms for further analysis.
